# On Moral Liberty

**Published:** 1857-10-01

**Authors:** 


### Art. III.—ON MORAL LIBERTY.

> "Heaven doth with us as we with torches do,
> Not light them for ourselves; for if our virtues
> Did not go forth from us, 'twere all alike
> As if we had them not."—*Shakspeare.*

WHAT is usually understood by Moral Liberty?

The brief answer is that man is a *free* agent. And when a more elaborate definition is given, it is to the effect—" that his actions result from a choice of different motives; and that, if *sane*, whatever may be the motives which may urge or warn him against or for an intended act, that this power is in himself, so that he can freely choose without any extraneous influence. Farther, that this power of selecting between different motives he has derived from his inherited privilege of moral liberty."

It is admitted that if he were always to act rightly and obey his better principles, that he would then experience a conscious satisfaction irrespective of any ulterior reward; but if he should submit himself to be the slave of the more selfish and animal impulses, let him

"Not lay the flattering unction to his soul"

that he can escape the certain penalty. For whether he obeys or disobeys the higher sentiments, he is still responsible for the consequences.

This definition, we apprehend, will be assented to as the reflex of the general notion of civilized man when reflecting on moral liberty based on *data* derived from a consciousness of his relative and positive duties.

But we would now ask an important question. Is moral liberty experienced in all men in an equal degree? Is this the case even when, *cæteris paribus*, their education has been similar, if not identical?

For the present we simply respond with a negative. But prior to offering proof as to the correctness of this opinion, it is essential to enter into some physiological considerations, beginning with a few brief anatomical facts.

At birth, the child is, organically, an imperfect being—that is, the general organism is immatured. The osseous system, for instance, is partially and in some cases entirely cartilaginous; the muscles want tone, and are soft and relaxed; whilst the brain, the nerves of the external senses, and the nervous-system in general, are in an immatured condition. This state of the different organs is but temporary, as provisions are made by the laws of our being for their ultimate maturity.

From the first dawning of existence, the Creator hath prearranged the means for this renovating process; the mother's milk is a bland, nutritious fluid, containing lime, fibrin, &c. These are the materials which furnish means for this renovating process, modified under the different circumstances; and as the process is uniform and under certain fixed and definite conditions, we designate the results as the "organic laws." We therefore premise that a knowledge of these laws is essential to comprehend the practical portion of this Essay.

It is also essential to keep in view the fact, that the brain, nerves, muscles, bones, skin, and so forth, possess the *vis plastica*, so that each system is enabled to appropriate such portion of the newly-formed blood as will ensure their respective development and growth. The tardiness or intensity of the power of assimilation is dependent on some original difference of constitution.

It should be also remembered, that besides this *elective* capacity of the different organs, that most of them are influenced by special stimuli, which become a cause and an effect in the organic process.

For example, *light* is a stimulus for the eye; and the tendency of this subtle agent is, to bring into activity all the organic apparatus essential to ensure unity of action in this marvellous optical instrument; and just in proportion as these different parts are affected, will be determined the quantity of the vital fluid required for their renovation. For in every part of our complex organism, great activity causes more blood to be assimilated, rendering each system more vigorous. So that with every improvement in the organic condition will be found comparative intensity of function.

Similar remarks apply to the other senses, and with their respective stimuli, *sounds*, flavours, odours, &c. With this glance at the *modus operandi* of the special growth of the organic instruments generally, we may proceed to a more important consideration, as connected with the special topic of this communication. And we may ask whether there exists any *data* to explain how far we are warranted in supposing that the instruments of the mental faculties (the cerebrum) are under the influence of the organic laws? And further, whether in such case the growth of the organs may be retarded or accelerated? That if an affirmation can be given to these questions, should we derive from this knowledge any positive advantage—particularly as furnishing some aid for elucidating our present psychological inquiry?

Before we could give any satisfactory answer, we must submit a few preliminary remarks, which we shall discuss under the following heads:

1st. What is the condition of the brain of a child at birth?

2nd. How are the mental powers strengthened and improved?

3rd. What are the kinds of *stimuli* which aid this development?

4th. Can any salutary and certain results be ensured, unless there is direct tuition under positive laws (psychological); and the special application of those laws by which all other organs of the body grow, acquire strength and intensity of function?

The condition of the brain at birth is soft, and the convolutions undefined. And this condition harmonizes with the imperfect manifestation of any mental process. Two modes of action tend to render the cerebral mass firm and matured, thus imparting a certain activity to its different organs.

The first condition is a rapid circulation of the sanguineous fluid, and equally rapid appropriation of the new material. The second results from the gradual development of the organs of the external senses, and the impressions made on them and through them to the brain; which impressions, by repetition, ultimately tend to call the perceptive powers into a state of activity.

And it is also judicious to keep in constant view the important fact, that each of the mental faculties (like the organs of the external senses) is affected by a distinct and particular stimulus. Just in the same way that the eye is stimulated by light, the nostrils by odours, the ear by sounds, the tongue by savours, and the surface of the body (touch) by the contact or impinging of any foreign substance, so also the perceptive faculties are affected differently; as, for example, the different configuration in all bodies is appreciated by *form;* that of various tints and hues, by *colour;* density, by our perceptive faculty of *weight;* and distance, by that of *size*, &c.

And just in the same way that one who is born blind could not appreciate forms and colours, so also without the organism to perceive, the mind would be incapacitated from taking cognizance of the qualities by which all material objects are individualized and distinguished. These general facts receive confirmation whenever there exists a malformation or injury of the lower portion of the anterier lobes.*

Similar remarks of the connexion between organic instruments and specific functions may be applied to the moral sentiments so far as indicating their strength or weakness, and also the laws by which they may be improved and strengthened. For instance, to stimulate the benevolent faculty, we must induce it to feel and act from a strong sense of kindness and forbearance. And the individual himself, during the period of his training, must be treated by those under whose surveillance he is, not merely with any mere wordy theory of goodness, but with that uniform and urbane manner which wins and attracts the young. And even in dispensing to the pupil all that may satisfy his natural wants, food, clothing, and other "creature comforts," he should be addressed in the mildest tones. A mere absence of harsh expressions is inefficient—there must be positively an agreeable and affectionate manner, a pleasing suavity, which forms the spiritual food for the growth of the benevolent sentiment; whilst rude and boisterous manners and rude taunting terms act like a deadly blight, and shrivel up this noble faculty.

* This is strikingly the case in what is called "Colour blindness." The defective power of appreciating or recognising particular colours is in the ratio of more or less deficiency of the organ of colour.

Similar remarks would apply with equal force to every other sentiment and feeling. If, for instance, a child evinces a cruel disposition, everything should be avoided which stimulates its action. Any sign of savagery in a parent or tutor tends only to induce a greater intensity of this power; and those who seem to delight in tearing off flies' legs may end, under a harsh and ferocious treatment in their own training, in thinking as little of destroying a human being, and feel no more compunction than in mutilating insects.

So also in the case of a child with great connate stubbornness, this being the abuse of a natural feeling implanted in the mind, but which if treated in a dogged manner, would tend to induce habitual resistance, and ultimately degenerate into stolid obstinacy.

These examples will suffice our present purpose. And yet there is another important practical fact which should be heeded by all grades of society—namely, that when we stimulate a mental power by either precept or example, *we stimulate the organization on which it depends for its manifestation.*

For the present we shall not press the subject, but simply remark, that he who recognises the absolute value of such *data* to those who educate, will admit that a knowledge of the *organic laws*, as applied to the mental faculties, is one of paramount importance, not only in training youth, but as suggestive of valuable and certain means for aiding the great object of philanthropists, the maturing a sound system of criminal legislation, which invariably should have for its object to raise the degraded portion of the community to a higher standard in the moral scale.

Yet prior to bringing forward the whole of our evidence with the view of establishing our views, and to prove that our ideas of moral liberty are based on accurately observed *data*, we deem it imperative to ask, whether any one who has studied the human mind under its diverse phases can affirm that all men are born with equal powers (intellectual and moral); and if we conclude they are not so, can we ascertain what constitutes the difference?

We answer affirmatively. And yet it is not any contradiction to state, that all mankind are similarly endowed, and that there exists but modifications in their different mental faculties. If there existed any absolute difference, then that would constitute a distinction of species.

We admit, therefore, that all mankind have similar mental powers, and our argument is merely as to the relative degrees in which they are manifested. We regard it as possible to establish such connate differences, whether we try the experiment with a family, a district, or a country. By the examination of the members of a single family, for instance, these constituting a small community, we perceive, however contracted the circle, differences of temper, disposition, and intelligence. And these variations could be distinctly traced to some modification of their cerebral organization.*

In addition to these remarks on the special differences which may exist, so also there may be observed a great difference in the general intellectual capacity of members of the same family. One, for instance, may possess such quickness of perception, that knowledge seems in his case to be intuitive; whilst his brother, more patient and plodding, shall be regarded as a dull and stupid boy. It would, therefore, be unfair to exact from each precisely an equal amount of progress in their respective studies; and if this were attempted, it could not be realized. It would be found just as impossible, as if we desired uniform results in tempers and dispositions of persons whose affective faculties presented the most opposite extremes—as, for instance, one who always deals in hyperbole and extravagantly exaggerates; and another who never compromises the simple and unembellished truth for either effect or applause, and who never utters a harsh expression when he has been the subject of misrepresentation. Could any training render these characters exactly alike in every particular?†

These, and numerous other differences might be indicated, and which from the dawning of consciousness were manifested as individual differences, and therefore could not be referred to any specific education. We admit that it is possible to stimulate the natural powers by judicious training, so that they may unfold all their latent intensity: and that when we attend to the natural laws (which combine physiological and psychological influences), we are furnished with a power to direct, modify, and restrain any excessive development of the whole brain on its separate faculties. The obvious advantage of this knowledge will be manifest as we proceed to unfold the practical part of this Essay.

In this place we may, however, remark, that if such differences may be observed in one family, we may anticipate still greater

* One may have a capacity for languages, another for music, a third for drawing, a fourth for mechanics, a fifth for mathematics, &c.

† We select the examples observed in one family. One was kind, considerate, and forbearing; another irascible and firm; a third orderly in all things and neat in his person; whilst his brother has not the least idea of arrangement, and never manifests any symptom of annoyance if all things were in a state of confusion. One of the family is extremely generous, and the other just as covetous. Another of the same folks will scold and storm on the slightest occasion, whilst his sister, even when greatly provoked, will manifest an angelic sweetness, as if she could not help feeling sympathy for the erring or unfortunate, and so forth. How in such cases can uniformity be anticipated?

diversity on a larger scale, as in the case of the inhabitants of small or large towns.

When we have carefully investigated the *data* furnished by patient observation on all grades and conditions of the community, we shall be prepared with such incontrovertible evidence, so as to deduce the important fact that there are different degrees of moral liberty; or, in other words, that all persons are not equally responsible for their actions.

We cannot be so unphilosophical as to suppose such differences to be accidental? They must be the result of some efficient cause. In the instance of the members of one and the same family, such must be the case; for the children are born of the same parents, and they are all surrounded by similar models for imitation and example, and with similar forms of direct tuition. If then each is still individualized, what other inference will explain the enigma, if we reject that palpable evidence we have deduced, that all these varieties in *disposition*, *temper*, and intellectual capacity are simply the consequences of some modification in the cerebral organization of each member of this small community.

Reject this explanation, and then it is obvious that we must continue to regard these plain and well-defined differences as something anomalous, or actually mysterious.

However, we affirm from long practical experience, that there is an immense advantage derived from positive knowledge on this subject; and especially so, when we know the mental constitution of each individual whose mind we might wish to influence or improve. It is a vast power, whether possessed by the parent or teacher. For this information gives a practical tendency, by enabling those who have the training of children or the correcting of the vicious habits of adults, to render their theoretical views not opposed to their daily acquired experience.

Hence, these conclusions seem inevitable from these premises: 1st. That the primary difference in the temper, disposition, and the comparative intelligence of persons results as certain modifications in the organic instruments of the mental faculties. And that, therefore, it will depend on their relative weakness or strength, the degrees of moral liberty which different individuals may manifest. 2nd. That if persons of all grades of mental power are to be benefited by education, the system must be primarily based on some definite principles, in accordance with our knowledge of the organic laws. 3rd. That in these deductions there is not anything speculative. For it is now ascertained beyond any doubt, that when persons of any inferior mental capacity derive benefit from education, it is noted that the improvement will be in proportion to the improvement more or less in the cerebral organization, which in extreme cases presents most marked differences. So much so, that casts taken of the heads of persons in a low condition of mental power, it is ascertained that when they have manifested greater capacity, if other casts are then taken of the same individuals, the two casts would not be recognised.

We are tempted to offer the following proof of the substantial accuracy of the previous statements, preferring it to others which we could submit. When the Idiot Institution was contemplated by that excellent and philanthropic gentleman, the Rev. Dr. Andrew Reed, he called on us, and we lent him a MS. copy of a work on the subject, mentioning at the same time our own experiments—saying, "Whenever there was observed any marked change in the temper, disposition, or intellectual capacity, and that these previously irresponsible persons began to give proof that they had acquired more self-control, corresponding to their improved intelligence; that whenever this was the case, there was also observed an alteration in the forms of their heads commensurate to this moral change." The Doctor seemed to regard the statement with some scepticism. But some years after the Idiot Institution had been established, the Doctor met us in London, and after a friendly greeting, said, "He had to apologize for doubting about the head altering, for the cases in the Asylum which had positively benefited by the system, that their heads had undergone a decided alteration, being greatly improved in form." After thanking the worthy Doctor for his candour, we affirmed that it afforded no surprise that such should be the case, as the organic laws, like all the other laws of God, were constant.

We make no apology for this digression, as it might naturally have been supposed, *à priori*, that such changes must be dependent on changes of the organization. For we could not have inferred that the immortal soul was subject to a state of idiocy or insanity, but that the organic instruments were defective or injured, those portions of the cerebrum by which the various mental faculties are manifested; and that the form of the head, in extreme cases, like that of a well-marked physiognomy, might give external indications of the comparative intelligence or stupidity; or of high and noble principles, or of sensual depravity—that these differences would be indicated so palpably, that the character of the possessor might be read with accuracy, and more or less appreciated even by those who may be unacquainted with any particular theory, but whose conclusions would be a result of intuitive perception.

A review of these general truths will warrant the inference that free agency must be regarded as being possessed in different degrees by different individuals. And although the conditions on which this difference depends may be traced to some modification of the cerebral instruments, let no one form a wrong conclusion. This knowledge is indeed a great power, for we learn that the brain, like the muscles, may become improved in volume and in intensity of function, by systematic exercise; and that it is also a truth, equally in reference to the organs of the mind and to the instruments of motion, that parts which are neglected and inactive shrink up and are less susceptible to their respective impressions or stimuli. So that by this knowledge we are furnished with certain, rather than by mere empirical, methods to improve mankind.

And this consists in using means to excite an active condition in the weak organs, and to leave the stronger and more impulsive powers in a quiescent state. For the cerebral instruments, like the muscles, will appropriate the vital fluid in the ratio of their active exercise.

If we had merely intimated our conviction that there existed a difference in the *moral* sense (conscientiousness), and that therefore some were more answerable for their conduct than others, there would not be any demurrer; because the fact is itself admitted by every civilized state, whose laws make a distinction between crime and disease. Hence the insane and idiots are not regarded as responsible beings, for in the eye of the law they are *non compos mentis*, a decision as sage as it is just—because idiots, with their defective anterior lobes, have not sufficient intelligence to regulate their own acts, and the insane, whether from fevers, injuries of the brain, &c., having so affected the condition of the mental faculties that the power to control mere impulses is lost; so that if either commit an homicidal act it is not treated as murder, but is regarded as the result of some illusory impression, depending on an abnormal condition of brain.

But with these enlightened distinctions, legislators have seemed to be satisfied. It may be true that they have obtained much information on the condition of the criminal-minded from the writings of medical psychologists. This knowledge was available for the purpose of rendering a distinction between disease and crime; but withal there still exists in the minds of the public many discrepant and uncertain notions of the subject.

There have also been made numerous statistical tables on the proportion of criminals with or without education. But what have been the practical results? Why, humanity has still to blush at the defectiveness of criminal legislation; which, whilst it attempts to apportion the penalty for each offence, has made little or no provision, on a scale commensurate to the exigency of the case, to provide means to stimulate the dormant powers of the *pariahs* of the community.

We may pause to inquire whether we have any *data* to explain the sources of criminal acts, and then discuss how far these evils are the necessary results of the constitution of man and the arrangements of society. And further, whether it is possible to do away with the evil, or modify in a great degree its virulence.

For our practical view of the subject, criminals may be classed thus:—

1st. Hereditary tendencies.

2nd. Ill-balanced organizations, which are easily influenced by evil associates.

3rd. Persons with naturally good or bad tendencies, suffering from poverty and neglect.

If we examine the history of persons born with criminal tendencies inherited from one or both parents, we shall find this tendency written on their brow "like the mark on Cain," and that, under the unpropitious circumstances in which they are placed, the tendency to evil is fostered even from infancy. Poor things, they ultimately prove "that it is not good for the soul to lack knowledge"—particularly a knowledge of God and their religious and moral obligations. All the teaching they have is addressed to the animal appetites, which by repetition, theoretically and practically, leaves such unfortunate beings more and more liable to sink into still greater abasement, from being less capable of resisting the temptation of morbid desires for sensual gratification. Where then is their free agency? How has society treated them? Are they not left surrounded by constantly exhaling moral malaria, which deadens all that is lovely and elevating, and leaves their better faculties in a state of hopeless torpor. We are speaking now of children, who will in their turn become the festering sore to injure the more industrious and worthy portion of the community. What otherwise can be expected? For, like their progenitors, they are instructed from their very infancy to become willing slaves of the lowest propensities. Initiated in lying and theft as soon as they can lisp, and suffering either from excess or deficiency of nourishment (both conditions acting as stimulants for animal cravings), they become confirmed criminals, acted on by the strong instinct of self-preservation.

The fear of punishment may at times deter them from an overt act of crime; but under strong temptation, even with the almost certainty of conviction, the consequences are unheeded. They lack any higher motives to restrain them, whilst the hope of impunity often renders them reckless. Under every varying circumstance they are liable to yield to their strongest impulses, without even the slightest compunction.

But let us remember that they have been taught to do wrong, and never how to avoid it. And their evil training has impressed them that *not* to succeed is a weakness, and that the only error they should guard against is not to be detected.

If we compare such early-trained criminals with the better organized, particularly when the latter have been carefully and judiciously educated, they almost form a distinct species. The moral liberty of these degraded beings is so low that it resembles more the imperfect kind manifested by such irrational animal as the dog, than those who are

"A little lower than the angels."

For instance, if one of the canine species steals a piece of meat, under a keen sense of hunger, and he is beaten by his master for the act, he is impressed with the fact that there is some connexion associated between the punishment and the "dainty bit," so that the animal will, under precisely similar circumstances, bear with the pangs of hunger, from recollecting the castigation he had received, and will thus avoid the repetition of the offence. In such a case he would, so to speak, choose between the motives of either eating and being punished, or avoid the latter and abstain.*

This, we grant, is a low state of free agency. Nor would we place it in juxtaposition with the moral liberty which is alone the province of man; but we have only quoted it to strengthen our argument, and to prove how low and degraded have fallen so large a portion of the human family, who have retained, in a vast many instances, but the outward form of manhood. Even such should be treated as if their *latent* powers might be cultivated, for all are born with a conscience or moral sense; and, although in the most degraded its voice is feeble, too much so to act with a monitorial influence, yet, unless we except the worse kind of idiot, it is possible, with a system of kindness and firmness, to stimulate it into a state of activity, and impart to it sufficient strength to restrain mere unmollified selfishness.

But to realise such comparative improvement, we must take such beings from the haunts of temptation, and place them under the *surveillance* of good persons, whose example and precepts might influence them. They should also be exercised by wholesome manual labour, but not to excess. They should be lodged in clean rooms, and fed on simple food. They should also be instructed, in their leisure hours, in some practical handicraft

* This example we cite from memory, as it was enunciated by the late most excellent and highly gifted Dr. Spurzheim, and it has, in our estimation, a great importance as an element of the subject under consideration.

knowledge; and if praised for every effort they might make for self-culture, then indeed "their sorrow would be turned to joy" with their first experienced sense of self-respect, and the sympathy and approbation of the better-disposed. On the contrary, if such efforts are neglected, let it be remembered that the repletion of vicious acts tends to deaden the sensibility of the *moral* sense, and like a cicatrized wound, it may be rendered altogether callous.

We are tempted to cite a criminal case, which will confirm many of the previously-stated views, and explain some of the predisposing causes of the social evils so injurious to the stability of the community. So that, whilst we shall be forced to acknowledge the wrongs of a large and abandoned class of our fellow-creatures, we trust that it will appear obvious that there is a positive necessity to attempt to reform them. For whilst there exists a large criminal population, it is a reflection on every God-fearing philanthropist, and a "mockery and a bye-word" on our defective legislation. For it is insufficient to enact laws for inflicting "pains and penalties" for criminal offences; it is an imperative duty not to rest satisfied until preventive means are matured.

With these remarks we will submit an example. A few years since a young man, aged nineteen, was executed, with his companion in crime, for a most atrocious and cruel murder of an old woman, who resided at a toll-gate in the suburbs of the metropolis.

This criminal, whose name was K——, was proved on his trial to have been most deplorably ignorant, and thoroughly vicious in his tendencies. His father had been a hardened and most reckless burglar, and a most inveterate drunkard, and otherwise a very depraved character.

What then could be expected from the son of such a man, who had had the bad example of his progenitors from his very childhood, and had only associated with similar worthless characters. Poor fellow, his only school was the pot-house, and his teachers mere reckless criminals and blasphemers. As he grew in years, it was but natural that his propensity for crime and for animal indulgences should greatly increase. Thus he frequented daily the lowest haunts of vice, and stimulated his powers for evil with strong and potent liquors. Theft was his regular occupation, and drinking to excess and other sensual indulgences his constant and never-varying relaxations. Uninstructed either in religion or morality, he knew not God or the duties he owed to society, whilst his intellectual culture was confined to facilitating means for rendering his nightly marauding successful. What then could be expected from such training, otherwise than that he should become a thoroughly depraved being, always craving to gratify his sensuality.

Thus much for his history. Let us examine the evidence which may in some measure explain it.

The writer has his skull. When first observed, the forehead appeared "villanously low," the anterior lobes being very shallow. When one of these lobes was measured from the spheno-temporal suture to the external anterior surface of the cranium, it did not exceed an inch in depth, whilst the base of the skull was large and broad, and the occipital region, taken from the *meatus auditorius externus*, out of all proportion; indicating on phrenological *data* a mere degraded organization, which would be confirmed by those who are acquainted with the anatomy of the skull.

And yet this head of K——'s would have been a case which in some measure would have proved the fallacy and erroneous inferences of mere "head-feelers," who, like all other charlatans, are too apt to judge by merely empirical rules, arising from defective theoretical knowledge. For, on looking at the upper part of the skull, there was a flat, table-shaped appearance of the sinciput. It did not appear at all like the form of head usually observed in such depraved criminals: a form which, when viewed from the mesial line of the upper part of the skull, appears shelved off on each side, resembling the gable ends of some old-fashioned houses.*

In this instance, the cranium of K——'s, if taken as a whole, would not have seemed so very bad. For many men with no better formed heads, who had received a moral and religious training, might have passed through life with comparative respectability.

Irrespective of mere empirical theories, there was a new revelation, by means of this skull, of a painful yet important kind. For when the calvarium was removed, it was a lesson it was not easy to forget.

The skull, on being sawn open, was shown to be more than an inch thick at the anterior and upper portions, whilst the base and the posterior region were so thin as to be quite diaphanous.

It must therefore be kept in view that this is not the appearance of a normal state of the cranium, particularly in a young

* Our dissent from the professional examiner of heads is this, that those who admit the accuracy of Gall and Spurzheim's theory must confess that all that could be inferred from the form of the skull and its different parts, is that there is merely indicated certain tendencies. But it would be injudicious to speak definitely of such tendencies, as much might have been done to modify them by systematic training.

and strong man; for if the brain and all its parts are uniformly exercised, there is a parallelism of the tables of the skull, or, in other words, a uniform thickness in the substance of the bones, save and except under the temporal muscle (the squamous portion of the temporal bones) which is accounted for by the constant working and motion of the temporal muscles over these parts.

What, then, is unfolded in this instance? Why, that which should make

"The unco gude and rigidly righteous"

blush that such a being should exist in a religious and civilized community. And let it be impressed in burning characters on those whose duty it is to prevent such persons from being utterly abandoned, how great is their responsibility. To leave such an individual under a morally putrefactive influence until utterly depraved, and as a consequence certain to violate the laws—then to be seized, committed to prison, and brought to trial—to be condemned by offended justice, and sentenced either to be hanged or sent to a penal settlement, where every flower-spot of the soul is obliterated, must outrage all notion of brotherly love, and all our affected solicitude for our erring fellow-creatures. We say then, fearlessly, that K——'s skull is more impressive than a sermon, and demonstrates this opinion through the evidence of the "organic laws." These have left indelible witnesses of his utter neglect and degradation. They stand as the accusers of society, which had permitted such an instance of saturated ignorance and crime to have existed, without any effort to save him (or his class) from the certain penalties inflicted on the violation of its laws. Nay, what is more, they knew not, nor cared aught for him, until he had outraged humanity and was punished.

But this state of things cannot be unheeded with impunity. The crimes committed by the K——s of society re-act on its members—for such criminals repay on them for the degradation of their position, by wreaking vengeance on some of the best and often the most innocent, which can only be regarded as a species of retributive justice for leaving them uninstructed, and for having avoided doing what might prove a painful duty, the earnest endeavour to correct their social and moral condition, and by such means save them from the inclination to, much less the commission of, crime.

Let us, however, explain how these "organic laws" may be admitted in evidence, to explain how they indicate the course of criminal abasement, and act as unmistakeable accusers of those who failed to devise means for instructing the people

We have already said, that when the whole brain is exercised the skull presents a uniform thickness.* That when parts have a disproportionate thinness, it is a proof that the cerebral organs in that region have been unduly exercised; and that, on the contrary, if some parts have more than a relative thickness, it indicates an inactivity or want of exercise of the organs in that region—the skull being, under these conditions of the brain, but the *index* of results: for, as under the influence of the "organic laws," the different organs of the brain assimilate new matter in the ratio of the activity manifested a neglect of any part tends to diminish its activity, and the organ degenerates in size and in intensity of function.

In the skull under consideration we have the most convincing evidence. Let us compare its appearances with what is known of his pursuits. He had never received any positive education of the higher attributes of the mind (the religious, moral, and intellectual), and it is found that the cerebral organs by which these attributes are manifested, shrank and shrivelled up, scarcely retaining any sensibility, and this loss of substance was compensated for by an osseous deposit. On the contrary, we have evidence of facts that the animal propensities were unduly stimulated, and that the posterior lobes and cerebellum had increased in bulk, and corresponding portions of the skull were, *ceteris paribus*, much thinned; in the latter localities *the inner tables and diploe were actually removed by absorption*, and the whole skull was the silent witness of a being wofully abandoned by teachers of all kinds—teachers who should have imparted to him real knowledge of human duty, and thus have saved him from crime and its consequences. But, with sorrow be it spoken, he was left to the searing and destructive influence of the lowest propensities, and sank into a premature grave, with scarcely a touch of sympathy for his miserable fate.

Let the pharisaical talk to such a culprit of human brotherhood, and of the sinfulness of vice—he may hear these words, but to him they would not impart any definite meaning. All that he actually knows is the fact, that in his own experience he has verified the Arab's character—"his hand has been against every man, and every man's hand against him;" and hence he has not any sensation of remorse, nor any perception of his wasted powers!

The civilized world is also cognizant of the fact that corporal punishment will not correct vicious habits. And, alas! it is also

* For the non-professional reader, we may state that the skull is said to consist of three layers or tables—viz., an outer table of dense bone, and an inner table of the same kind; but between these tables there is interposed a softer and spongy substance, called the *diploe*.

proved that capital punishment does not prevent overt acts against life and property.

We must go to the root of the evil, and if possible destroy the seeds of crime. For if they are permitted to remain in the incipient stage, time will mature them—they will fructify, and develope their fearful energy, often to the injury of the most harmless and the most worthy.

To succeed in effecting any reformation, encouragement should be given to induce the idle and worthless to take to honest labour. This should be done at a great outlay, which would after all be a thrifty investment, if it succeeded in forming industrious rather than criminal habits. And on striking a balance of the present cost for gaols, workhouses, police corps, penal settlements, and judges' salaries, there would be left a surplus in favour of this reformatory plan.

Besides, there might be instituted, in all localities, certain prizes to be given to those who could furnish evidence of having given up a career of evil, and made some improvement in mechanical or handicraft knowledge. The advantages are many —not only the desire of improving their own worldly condition, but also a certain satisfaction from the kindly approbation of their conduct by the better cultivated grades. Of course in this experiment we allude to the children of the criminal population; with their progenitors there is something else required. There is with adult criminals the absolute necessity to prevent their drunken habits; then there might be a chance to render some of them better. With sobriety, they would be more disposed to listen to advice, and be more likely to try and live by their own industry.

Judge Talfourd, at his death, bequeathed a sacred obligation to the fortunate and moral portion of the community. He feelingly and truthfully pointed out, in that brief address, the great advantage which would result by occasionally mixing with those who were degraded by their position or their conduct. He pointed out the benign influence of this giving friendly counsel to the ignorant, and to those most liable to temptation; persons whose minds are blankless, and who therefore neither appreciate the marvels of the outer world nor the sources of endless satisfaction from cultivating those spiritual perceptions which connect themselves with our duties in this life and with our eternal hopes.

In the case of the criminal K——, what was it that had been done for him, or what had been tried to save him? His nature was perverted, but it was from mere stolid ignorance. How could he be expected to appreciate the beauty of moral science, or the profound pleasure to be derived from intellectual operations, when he, poor fellow, had never been taught "the way he

*should* go," or else he would not have departed so fearfully from the right path.

But when every incentive to good had been destroyed by all that the animal appetites could command, he naturally became the slave of vice, and would have continued in a career of crime had not the law forcibly interposed, and demanded his life as a sacrifice for his wicked and sanguinary deeds.

Still, let the humane pause ere they attribute all blame to the criminal, whose acts were indeed but the natural consequences of his neglected condition. We then desire to see matured some plan to prevent the development of criminal tendencies, else it would be indeed a waste of time and thought to treat on such subjects. Nay, those should blush who, after contemplating the evil as it exists, with all its ultimate consequences, yet remain supine when the work of reformation has scarcely commenced. Is it not the duty of all who possess the least influence to aid in arresting the increasing tide of moral desolation—a desolation which must ultimately swamp and lay waste everything good among the community?

Can we continue to suffer so many of our fellow-creatures, particularly the juvenile portion, to be steeped in sin, and find excuses for ourselves for not making a strenuous effort to prevent this continual outrage of the laws of God and society?

We know from the history of a vast number of cases similar to K——'s, that such beings are regularly initiated in every species of vice from their very infancy, and that the only precautionary lesson they are taught is to avoid being detected when pursuing their dishonest practices. How can there be expected any moral nausea, when it is known that their successfully-performed criminal acts are boisterously applauded by their hoary and debased teachers; and are encouraged and stimulated by their peers, under the evil influences of gin and beer!

Many criminals are actually ignorant of the laws, and consequently do not consider anything they do any positive violation of them. So that all they think of as essential to themselves is abiding by the laws of self-preservation.

And now we ask, in sober earnestness, can we consider persons so neglected and so circumstanced to be possessed of a similar degree of moral liberty as those who are placed in more auspicious conditions?

We shall next briefly discuss our second proposition—namely, The effects induced by neglect and poverty, acting on either a naturally bad or good organization.

When the better educated classes, who are possessed of ample means, reflect on the necessity they feel for change, and for various relaxations, merely to avoid the misery of *ennui*, they should then feel great sympathy for those whose very monotonous employment induces such a jaded condition of the nervous system that they have recourse to less pure means to ensure counter-irritation. This result they fancy they obtain by sotting themselves in pot-houses, where they only stupify their intellect and brutalize their passions. Others may combine the hope of gain with amusement, as in poaching, the excitement of which is often more stimulating than strong drinks. We allude to these latter cases, in order to point out the insidiousness of crime. For there are on record many instances where men commenced with poaching, and then gradually became initiated in other species of peculation, until they ultimately became daring midnight robbers or savage burglars.

Our legislators, cognizant of these facts, have framed laws which, for all practical purposes, seem to acknowledge the principle of different degrees of moral liberty. Although there are often great discrepancies in the way in which these laws are administered, and which might actually induce a supposition, that grades of crime are regarded only as true in theory, but not *ipso facto* so in practice. For, although there is a scale of fines and different periods of imprisonment apportioned for shades and degrees of criminal guilt, yet, in the application of these various punishments there is too much left to the discretionary power of the magistrate, who, being human, is liable, according to his humour, to be too harsh or too lenient. These errors of administering the laws result from not sufficient attention being paid to the condition and motives of the various delinquents.

We recollect a most interesting case in the Derby County Gaol, which struck us at the time with a variety of topics for reflection. Among which weighty considerations, it appeared that too often the punishment is more than commensurate to the special crimes, and that often a first offence is treated too harshly, tending in such instances to pervert the ends of justice. For it is intended that punishment should correct errors, and not convert that very punishment into a means for hardening an offender, thus making him a great plague-spot on the community.

The County Prison at Derby is built on the plan of Jeremy Bentham's Panopticon, being of a circular form, with a similar shaped "court-yard;" the different offices being arranged in *radii*, the governor's residence one of them—the advantage of which is, that this functionary can at all times take a survey of the principal parts of the establishment.

Between every *radius* there were narrow spaces, with strong iron-work gates. These places were the solitary cells. In passing one of them, our party were much interested by a picturesque-looking prisoner, who was pacing up and down the narrow walk.

On requesting the governor to give the writer of this permission to speak to the prisoner, he politely declined, saying that he could not do so without an order from a visiting magistrate. Fortunately, one of our party was in the Commission of the Peace, and he having intimated his willingness to grant us this favour, the massy gate was unlocked, and as soon as we had entered the precinct, it was again fastened. From the aspect of the prisoner it was evident that he was painfully conscious of his degradation, and that he nourished already a deadly hatred against society in general, and against those in whose power he was in particular.

To comprehend our actual position, we shall attempt to give a sketch of the scene. The space of the miserable path, where we stood face to face with the prisoner, just admitted a full grown man to walk up and down; whilst the length of the promenade itself did not exceed eight feet. At its upper extremity was the solitary cell, and at the lower end stood its sole possessor.

When we first entered, the prisoner's brow was corrugated, his fists were clenched, and his whole expression marked one who was altogether desperate, and whom to irritate might endanger one's life. As we had neither motive nor interest in offending him, although he silently scowled upon us, we did not experience any sensation of fear. We spoke to him in a kindly tone, and expressed great sorrow that such a man should have his liberty abridged: for he was a fine, handsome-looking fellow, with a good head, and a rather naturally intelligent countenance. We unfolded, in a few brief sentences, our notions of the human family, and then explained the source of our sympathy, that any one should violate the laws, and forget the ties of brotherhood which bind men together for their mutual good, and for protecting each from any selfish aggression. We do not wish to repeat our homily. But suffice it to say, that gradually the muscles of his face and hands relaxed, and tears started in his eyes. He stood like one metamorphosed from the savage state to be under the influence of more humane sentiments. The change seemed marvellous. And, as he put out his hand to shake our proffered one, he said—"You, sir, have made me do what I have not done for years—shed tears, like in my boyish days; for I have for some time past only nourished feelings of hatred and revenge for the wrongs which have been inflicted on me. I never thought that any one could or would ever show me so much kindness," or words to the same effect.

We were then curious to know something of his history, as he seemed one suited to a better fate. We therefore said—"Will you tell us for what act you are deprived of your liberty?" There was another frown passed over his fine features, but it soon passed away.

He then related his case, which was a too commonplace occurrence. He had been guilty of poaching, and had been committed to gaol for a long period; and as he was punished so severely for his first offence, he determined to be revenged. In the prison he found many hardened and adept teachers of crime, who encouraged him in his proposed intention. On his liberation he repeated the offence, and was again incarcerated.*

The effect of these repeated punishments was frightful on the mind of one who had but the rudiments of education, but who had natural powers which, had they been properly fostered, would have rendered him a benefactor rather than a tax on the community. Our experiment confirmed this latter view of the subject, and proved how much could have been done by the magistrate who punished and degraded him if that functionary had acted on the law of kindness: he would have saved an erring brother, and given another instance of the omnipotence of the *law of love.*

The Derbyshire poacher was evidently a man with naturally strong moral susceptibilities, and in his case, the appeal to them would have fostered all the elements of good in his mental constitution, whilst harshness addressed merely his animal propensities, and induced in them a fearful intensity.

We therefore contend that, to make all punishments correctional, they should aim, in the first instance, to prevent individuals from doing evil by especially working on the higher attributes of the mind: in other words, the object should be to train the *latent* better faculties. And those who have been left in ignorance, not having, therefore, any positive free agency, should be treated as children, by being prevented from doing wrong or getting into mischief, whilst they should be systematically taught what is right and what are the respective duties of all men in society. They should not be permitted, in their ignorance and depravity, to violate the laws, and thus suffer the penalty.

It is admitted in theory that all punishments, whether for children or criminals, should not have anything like vindictiveness, but should be strictly reformatory. For instance, instead of sending persons for their first offence to a prison, a reformatory school should be selected, as in it a better spirit might be awakened; but in others, as a gaol for instance, it generally proves to be a college for fostering crime.

* We forget whether or not he had been transported for his outrage against the game laws; but we well remember how he regarded his treatment as unnaturally vindictive.

We consider punishments which are almost unproductive as doubly injurious. If, for example, instead of the treadmill, and similar wearying occupations and waste of muscular power, there could be substituted some useful and profitable labour, giving, at the same time, a certain number of hours to mind-culture, then some lasting reformation might be anticipated even among adults. But to be worked merely to produce painful inconvenience to the offenders, and without any higher motives for moral respectability being enforced, and then to be locked up like savage and untameable animals, must necessarily blight every latent better sentiment, and send them to society improved in lessons of evil, and with a determination to commit more aggravated offences.

We may be told that all these evils are known and lamented, and that various remedies have been tried to alleviate the virulence of these diseases of our social system, but without producing any permanent effect; that when once criminal desires take possession of certain minds, whatever palliatives are proposed have hitherto proved to be inefficacious, for it is asserted that crimes of all kinds are epidemical. And there are those who stand stoically and contemplate these evils, "which cry aloud to heaven," and exclaim that there is no help, for this is

——— "a world of human wretchedness,
And a world of human strife,
Sorrow, and wrong, and weariness,
That began and closed with life."—*Tinsley*.

But various remedies have been proposed, and amongst them "the silent system" was deemed the one which should work wonders. It was said, that if criminals were not allowed to speak to each other, the evil-minded could not corrupt a mere temporary offender. This may be partially true, and yet is not any cure for the evil. To improve beings who are endowed with intellectual and moral perceptions, so that they may comprehend their actual responsibility for all their acts, it must be evident that negative means will prove altogether fallacious. What is actually required in all such cases is, that the mental faculties should have positive cultivation, and their normal condition attempted by a system of active and agreeable exercise of these powers; stimulating hope that brighter and calmer days will be their reward for every effort they may make for their own self-improvement.

When the "silent system" was first tried in this country, the House of Correction at Wakefield was chosen for the experiment. Being desirous of knowing all things from actual observation, we proceeded to that town, and were accompanied by the excellent and intelligent surgeon of that prison, to ascertain for ourselves the effect of the new discipline.

In the work-rooms of the men there appeared the stillness of the grave, so far as the absence of the sound of the human voice warrants the comparison. The overseers in each room were themselves criminals; but their post, as spies on the actions and conduct of their fellows, was rewarded by a remission of all manual labour, and by a more than usual allowance of a better kind of food than the ordinary dietary of the gaol.* Animal motives were the only ones addressed under this system, as they had been under every other; for these overseers were held to their rigid duty by the idleness and comfort they obtained, and by the fact that, if they allowed any of the prisoners at work to interchange a few words, the reporter of any such lax discipline on the part of an overseer would lead to this consequence—they would exchange places; the overseer would have to take the work of the informer, and the latter would assume the rank and invidious position of his predecessor.

It was something fearful to contemplate so many *automata* working without a purpose, with scowling faces and vacant minds; and when they marched in gangs to their dinner, making no other sound than the slight one produced by the motion of their bodies, with their pale and jaded faces unmoved and uninterested, it was like a procession of the dead, literally and positively chilling us.

We then visited the infirmary, in which were many degraded organizations; but in one bed near them there was a good-looking, kind-hearted youth, who seemed of a distinct species from the rest of these patients. We remarked to our benevolent companion that it was a pity such a lad should be placed amidst and associate with such contaminated companions; but the answer was, "They must not speak, so they cannot corrupt him." The offence of the youth was that he had run away from his apprenticeship.

In the female department there occurred an incident which induced us to think that one of the reforms in the treatment of criminals must be classification, and subsequent experience confirms this imperative necessity. In the work-rooms and wash-houses there were many debased-looking, low-browed, broad-headed women, with all that harshness of features and slovenly appearance which is an invariable result of a long-continued wicked course of life. As visitors, we could speak to any of these unfortunate creatures. We asked some of them a few questions; it will suffice to state the answers of one of them. She was an uncouth-looking person, and had not anything feminine in her personal appearance, whilst the coarse habiliments of the

* This remission of labour and all task-work took place even when hard labour formed part of the sentence of these overseers.

prison and cropped hair, her coarse dissipated features and husky voice could not for a moment induce us to associate her as one of the "fair sex," for drinking and low habits had impressed on her visage a most forbidding expression.

To the question, "Have you been in prison before this time?"—"Oh yes, many times."

"What number of times, do you suppose?"—"Why, this is the seventeenth, so far as I remember. I may have been more, though."

This was said without the slightest symptom of shame, but on the contrary, there was a harshness of tone and a bravado of manner which demonstrated that the speaker did not perceive her most lamentable state of degradation.

What degree of moral liberty had such a criminal? She had not any desire to control her animal propensities; she never, at least not for many years, had experienced any of those exquisite emotions which spring from the aspirations of the moral attributes. Her principal delight was in gin, beer, or rum, and in the exercise of the lowest animal functions; whilst the latter reacted on her mind, warped and almost obliterated all feminine notions of propriety, rendered her indolent and dirty, with a distaste for all active industry. We felt shocked and horrified at beholding such a being, who was oblivious of all purity of thought and every refinement of sentiment.

After taking a survey of the other wretched specimens of humanity, in which the sample described resembled the bulk, we turned away and beheld in the distance a very different sort of female prisoner. She was a very young and good-looking girl, with an excellent cerebral organization. She was standing alone and in a pensive attitude; for, though a prisoner, she must have felt herself contaminated by the degraded party we had just left. Accompanied by our friend the surgeon, we approached this isolated person, and commenced by saying, "Surely, you are not a criminal?" The poor girl burst into tears and sobbed convulsively, and it was some minutes before she could answer. She stood before us as a strongly-marked contrast to the sordid and degraded women behind us, who appeared unconscious of their low and miserable position; she, on the contrary, looked like a beautiful Magdalen, as her contrition gave her a peculiar charm, and her sense of self-abasement represented her in a most interesting point of view, inasmuch that her conscious degradation gave surety that she desired to redeem her lost position.

A few words of kindness from us restored her confidence, and enabled her to reply to our repeated question. She then told us that she was the daughter of a non-commissioned officer; that she had lost both her parents in one week from typhus fever, and that, in consequence of this bereavement, she was left chargeable to the parish. That the functionaries had placed her, for cheapness, at the house of a low and dishonest woman; that she had never been taught to read or write, and that she never had had any knowledge of religion communicated to her; that this "foster-mother" selected by the parish authorities had instructed her in dishonest practices, often sending her with things, stolen by herself or her associates, to pledge at pawnbrokers; and that she was taken up when despatched on a similar errand, and committed because the stolen property was found in her possession. Her tears and sobs frequently interrupted her short and painful narrative, and excited our sympathy for her forlorn and unpropitious situation; for, although very illiterate, there were *latent* faculties of a superior kind which only required the fostering hand of culture to have rendered her whole being metamorphosed.

We remarked at the time that, instead of being sent to a common gaol, this poor creature, with her naturally good qualities, should have been sent to a reformatory institution, where she could have been trained to be a useful member of the community. There is nothing gratuitous or speculative in these notions; for although this young girl had been utterly neglected in secular knowledge, and scarcely knew anything of God or religion (if we except the few childish lessons she had learned from her own mother in her infancy), and with the glaring fact that she had had positive instruction addressed to her animal propensities—lying, theft, lewdness, swearing, and blasphemy—these lessons being communicated by example and precept, and yet she had not been rendered altogether callous, but her better sentiments still responded to any appeal to them. This was evinced by the scalding tears of deep contrition which welled from her heart when touched by the palpable sympathy of genuine kindness; whilst her degraded companions—degraded by their low connate organization and the abandonment of all culture—had neither a sense of shame nor any feeling of compunction.

Surely, then, we are warranted in our inference that the good-organized possess, under ordinary circumstances, more actual moral liberty than those who are naturally defective in intelligence, and suffer also from their hereditary contaminations. And it is worthy of remark that, in any attempt for the reformation of criminals, this distinction must be strictly regarded. We might rapidly improve such a mind as that of the girl mentioned above at the House of Correction; for her own innate tendencies would appreciate kindly motives to improve her, and thus she would use her own volition to aid such efforts in her favour. Whiist the class of criminals represented by the other women, her accidental associates in the same prison, would require much greater labour and more patience in any attempt to reform them; and then their improvement would be limited, and rather partake of a negative result: that is, if such criminals could be kept in a House of Industry and induced to work for their own maintenance, and strictly forbidden intoxicating drinks, and at the same time if a little moral instruction could be given them, they might be prevented from continuing in a criminal career, but we could never hope to render them so morally elevated as to lose every vestige of their previous vicious trains of thought. We might palliate the evil in adult cases, but it is to the young that we must direct our most energetic attention.

If we make a strong effort with incipient offenders of the laws, there might be a chance of a permanent reformation; for if we commence our task before bad habits had become actually a second nature, success would reward our labour. But in those whose career of vice had seared every better principle, and rendered the *moral* sense callous, there may exist a reasonable doubt as to the sincerity of their affected improvement. When, on the contrary, there exists, as in childhood, an elastic spirit, and a capacity in their pliant faculties to take, by judicious culture, a different direction to any temporary distortion, we may calculate on realizing a certain amount of good. To render the experiment productive of such advantages, we must transplant such children to a more genial atmosphere than the noxious one of their own degraded parentage; and then we shall have the lasting satisfaction that we had raised up a number of human beings to a higher standard in the social scale, who must have inevitably, without such extraneous assistance, sunk to the lowest depths of vice. But those redeemed from vice will repay the outlay and become a blessing to themselves, and a benefit to the community which had supplied the means for effectuating this change in their condition.

But with all such efforts it would nevertheless be a Utopian scheme to expect that any system can be productive of such a reformation as to effect anything like uniformity in all cases under treatment. There was, for example, a natural and strongly marked difference between the girl and the woman in the Wakefield House of Correction. She, poor thing, was naturally well disposed until perverted by bad teachers, but who would, under better auspices, acquire perfect self-control, and an honest desire to learn and perform her relative and positive duties. But what could be expected from the one so organized that she could unblushingly proclaim her own worthlessness, and who could speak of her seventeenth incarceration for theft without the slightest attempt to palliate her conduct.

It is, therefore, in such extreme cases that we have indubitable evidence that there exists different degrees of moral liberty, so that free agency will be experienced in the ratio of the actually inherited character, modified by the neglect of culture and by the consequences of comparative abject poverty; and when even the natural powers are good, yet stolid ignorance and extreme penury are often predisposing causes of various kinds of moral delinquency.

Thus we infer that criminal reform, to be productive of success, must be commenced in childhood; yet we should deprecate any attempt to be made for this purpose in their degraded and polluted homes, although the brutalized parents should be made to pay something towards the maintenance and education of such embryo criminals. The principle is not a new one; for if parents do not do their duty in preparing their children to be worthy citizens of the commonwealth, the State must undertake the parental function. Many reflections were forced upon us from what we observed in the different prisons we visited, which bear on our present subject, but we shall not have space to go into details. We cannot, however, resist mentioning one fact—that the whole system of the treatment of criminals is a mistaken one; as every appeal is made to the animal propensities, whether to reward or punish, if we except the religious service, which from their defective knowledge they can scarcely appreciate. For living in open violation of the moral laws, and ignorant of their positive and relative duties, the beauty of religion is veiled, and therefore mere verbal descriptions convey but imperfect conceptions, from their incapacity to associate any positive ideas of the oral expressions used in teaching them. Man is a complex being, with faculties which fit him for his earthly labours, and moral perceptions to regulate his conduct; but if all his energies are concentrated on mere sensual gratifications, then it is injudicious to appeal to the animal propensities in any correctional attempt, and this is done when they are made to feel pain and annoyance, so that no actual advantage is gained. We affect to cure a malady, and all we do only aggravates the symptoms, and our treatment is altogether empirical. For it seems to have been forgotten by those who legislate, that whatever may be the degraded state of the criminal population, that they have mental faculties which could be trained, and moral perceptions which could be addressed. And if with such beings the process is slow, the results may be somewhat cheering.

It would require too much time to bring forward a mass of evidence in confirmation of these statements, and probably there is not any necessity to go into minute details, so we shall refer the reader to what was said of the method adopted by the framers of the "silent system." It will be remembered that the criminals selected for the experiment gained for themselves scarcely any experience to serve them when discharged from prison. They certainly learnt an additional fact—that better educated men had used their privileged positions to *bribe* them to do their duty. The bribe, although not in solid cash, yet only appealed to the animal, for it consisted in an extra allowance of food, and a state of bodily and mental inactivity.

Criminals did not certainly derive any permanent benefit from this system; they remained mentally inactive, and this supineness reacted injuriously on their bodily constitutions; and yet criminal reformation is spoken of as if it were something to be realized by a mere outlay of money. What reformation can be expected from rewards addressed to the animal? Why not make an effort to speak to man's moral nature, and to give him purer motives for his conduct. If rewards are essential, then promise him, if his conduct is good, that he shall not only have a good certificate, but shall have some honest employment procured for him. Such a plan would be productive of a twofold advantage, there would be a chance of the reformation being permanent, and an almost certainty of awakening in the mind of the reformed a sense of self-respect.

Taking the experience of the prisoners in the House of Correction at Wakefield, they must have regarded the conduct of the superintendents as mean, sly, and vindictive. For instance, it was regarded as a most aggravated form of offence if any of these unfortunate beings ventured to relieve their monotonous condition by speaking aloud in their cells at night, for they were punished for this contumacy—and the means of detection was made by the following method:—

In order to ascertain whether such offences were committed, the turnkeys wore list shoes, which allowed them to glide along the passages, not like guardian angels, but as invidious informers. For greater facility the corridors were lighted all night with gas, and woe to him who was heard to breathe out a complaint in audible language, even if it were a prayer to the throne of mercy. It was sufficient offence for these "watchers" to have heard the sound of words; for immediately they fixed the fatal proof of this audacity by putting a cross in chalk on the door of the cell. In the morning, one of the functionaries of the prison went his rounds, and on every sleeping apartment on which the fatal mark was found, the offender was brought before the governor, who, after hearing the statement of the official, sentenced the astonished delinquent to be deprived of half his breakfast.

This demand of the propounders of the system for absolute silence on the part of prisoners rendered *espionage* absolutely necessary, and should modify our verdict in judging the conduct of the officers of the gaol. Yet we cannot help deprecating the system, as opposed alike to the laws of our humanity and to the social and gregarious instincts of our race. It was the outrage on these instincts which had a fatal influence on the minds of many; for such as those who had to suffer, it is no wonder that they endured depression of spirit from a death-like silence; and though amidst the ominous stillness they might have felt morally nauseated because

"Of time misspent and labour lost,"

it should not have been regarded as a crime when the full heart gave vent to its feelings, and men sighed out an "Oh dear!" or "God help me!" yet for this symptom of moral convalescence they had given them a half allowance of food.

It will be also to our purpose to mention, that in this "secondary" punishment the animal appetite was made to suffer craving for food. But was this treatment calculated to render an individual morally better? How then can it be expected that men shall come out of prison, after having undergone their period of punishment, better disposed and more in love with mankind? What scintillations of a desire for improvement are annihilated by what would, to the mind of such persons, be regarded as unnecessary and unnatural harshness.

Alas! we treat prisoners, not as if they had common feelings with us, but rather as mere animals, and by these proceedings defeat the ends of justice. And when we reflect on the expensiveness of the machinery to restrain and improve this *pariah* class, and the fact that after the jail-treatment they become more vicious and vindictive, we can only come to the conclusion, that all preceding attempts to reform criminals have signally failed, because legislators have not understood the mental condition of such offenders.

Criminals should be regarded as imperfect men and women. That from defective or perverted education they have not any conception of the fitness or non-fitness of certain acts. Their mental vision is defective, and they blindly follow their strong animal propensities as irresistible impulses. How then can they be expected to take a view of human duty in that clear and unmistakeable light which men do who have normal minds?

Moral Liberty being derived in the latter case from the reflected rays of a cultivated intellect and moral perceptions, in such an instance, there is *true* free agency—that is, the high privilege of having the power to regulate actions. With such capacity for self-government, an individual becomes a benefactor to his species. Any one, on the contrary, with his higher mental faculties so torpid that he is the slave of his feelings, cannot comprehend what is meant by moral liberty, unless it be to do whatever he pleases. He has not an idea that it means the power by which we choose between motives, and by which we hold the rein on selfishness. Hence the criminal-minded are deprived of absolute free agency, as they injure others and themselves, and so impede and clog the wheels of civilization as to obstruct its workings, and prevent any permanent improvement in the constitution of society.

To awaken within this unfortunate class a sense of moral liberty, we must cultivate their dormant faculties, and teach them how, by respecting themselves, they will gain the esteem of others. These results might be attended with great, very great difficulties; but if we can teach the idiot, with his almost absolute negation of intellectual capacity, we may hope by similar patience to improve the criminal or morally-stunted being .At first criminals might not have a full perception of their own necessities, but they could act on this philosophy of the Great Bard—

> "Assume a virtue, if you have it not.
> The monster, Custom, is angel yet in this,
> That to the use of actions fair and good
> He likewise gives a frock of livery,
> That aptly is put on. *Refrain but once,*
> *And that shall lend a kind of easiness*
> *To the next abstinence; the next more easy.*
> For use can almost change the stamp of nature,
> And either curb the devil, or throw him out
> With wondrous potency."

And as a matter of much observation and experience, we would also enforce the truth—that when the novitiate in criminal practices renders it necessary to inflict on him some form of punishment, that care should be taken not to destroy "the sense of shame" in him; and we surely do so when he is degraded in a public manner.

It is said, "that first impressions are often the strongest." This is strictly so when some truth is presented so vividly, as if the voice of the Omnipotent had spoken it. The mind then instantly seizes it, and it is indelibly impressed. For whether this is applied to some moral principle or to some law of physics, it is equally tenable.

We are tempted therefore to narrate a *scene* from which we deduced many practical reflections on the treatment of a first offence.

Many years ago, when a young man, we were visiting C——, in the county of Essex, and being told that two lads were to be publicly whipped, we desired to witness what effect it would have on them, having had a presentiment that any such public degradation could only tend to debase the recipient.

And this conviction was suggested by reflecting on what would be the effect on a *sensitive* mind, and on one who had already lost all purity of thought and the desire for the approbation of his superiors. The sequel will show that these inferences were the natural inductions of moral laws.

On the morning of this painful exhibition there were a vast number of persons congregated before the gaol "to see the sight," and, although we were amongst them, we did not anticipate anything but pain from such a spectacle.

We were told by one of the by-standers that the elder of the two delinquents was a stranger in the town; but that he had been a groom to a gentleman who resided there, and had on the present and, as it seemed, the only occasion, been guilty of dishonesty, by selling some oats and corn belonging to his master. That he was sentenced to six months' imprisonment, and to be twice publicly whipped; once at the commencement and the other at the termination of the sentence. We must make a short digression, that those who convicted this criminal knew little of human nature, when they decided that he should commence his supposed reformation with a lacerated back, and morally smarting under the infliction of the disgrace. We need not make any comment on the school of proficient criminals this comparatively innocent young man would be associated with for six months, but merely remark that at the expiration of his sentence there was to be a renewal of the laceration, and then he would be turned out without means or character, with bleeding wounds and an irritated mind, and what but a repetition of crime could be anticipated?

The other prisoner had robbed, in a most daring manner, the shop-till of an old woman; and as he had been previously convicted, he had also a similar sentence. The prisoners were in a large cart, with naked backs. The stranger had on a cap, which he pulled down very low, to shade his face, on which there was an hectic glow. The other was without any covering on his head.

The stranger lad cried very much, but the *native* boy looked impudent and full of bravado. This was particularly the case with the latter when some of his acquaintances called him by name, and urged him to show "pluck." He shook his head in a dogged manner, and then a momentary smile passed over his features, as if he felt sure of sympathy in proportion to his indifference of the expected lashings. He afterwards turned to the other culprit, seemingly to urge him to show defiance.

The cart moved slowly, though drawn by a strong horse. It proceeded from the front entrance of the town-gaol to the top of the principal street, and then back again to the starting place. The functionary who whipped them stood inside of the vehicle, and he laid on in earnest.

When the cart stopped, it was a sorry thing to see two such young lads with their backs lacerated, yet evidently without the slightest salutary effect on their minds. On the contrary, it was obvious to every reflective observer that the whip had destroyed any *latent* sense of shame they might have had. The stranger was indeed metamorphosed. His cap was off, he was flushed, and had an expression of contempt on his lip. The native's face was brazen and savage, and his laugh was one of defiance ; and one might have read in the features of both something which indexed a long list of greater acts of criminal depravity.

The stranger, we understood, could read and write, but the native lad could not do either. What reformatory influence could be expected ? The very punishment damaged their moral nature, and thus prepared them for greater injury, as they now regarded society as their implacable enemy. From that hour to the present time we have deprecated public punishments for juvenile offenders, as inducing no kind of moral benefit, and only leaving a lasting tendency to brave things which is destructive to all chance of amendment.* And, acting on our own experience, we have urged on the attention of educators the erroneous principle of exposing the conduct of wilful children before strangers, as it is offensive to their self-esteem, and pains their love of approbation, and rouses merely their bad passions, but never can stimulate the moral sentiments—

"And men are but children of a larger growth."

The stranger, for example, who was publicly whipped at C———, could have been redeemed to the path of virtue by kindness, and by an earnest private admonitory appeal to his better nature, as there existed "a still small voice ;" in witness thereof recollect the bitter tears he shed of remorse or shame when first publicly exposed. Instead of such saving effort, after his first error, the constable was sent for, and he was taken before a magistrate, examined, and committed for trial. After this first searing process, he had to appear in open court, where again his misdeed was rehearsed before a larger assembly: this was the second searing process ; and finally he was brought out into the street with naked back, and publicly beaten, like an animal might be who had not any moral sentiments ; and this last act warped and destroyed his better nature. Further, there was betrayed by the dispenser of the laws his positive ignorance of the constitution of the mind, and of moral philosophy, otherwise he would have discriminated in the punishment awarded in the two cases referred to ; for it was evident that the stranger had a keener perception of his degradation, from having more moral liberty,

* The two lads mentioned above became adepts in crime. The stranger was hanged for forgery ; and, if our memory serves us, the other was transported for life.

than the native boy. As the first had had some education, which gave him greater power of self-control (free-agency), and some knowledge of his responsibility for his actions, this accounts for his being so deeply affected; whilst the perfectly ignorant and illiterate boy was so shamefully brazen-faced as not to feel the disgrace. A writer remarks—

"Until we know what we would be at, how can we come at it? Where is our Themistocles or our Lycurgus? The wisest, the best of the God-gifted of our race are scarce adequate for the achievement of penetrating the depth of human nature, dissecting the laws of its moral being, and detecting the sources and devising the agencies of its progress and perfectibility."

After showing the inability of official experience, he continues:—

"Mere talent or knowledge, administrative skill or worldly astuteness, will light a short way on this path. We have to deal with the infinitely complicated mechanism of this wonderful spiritual microcosm —its hopes, its fears, its fancy, its sentiments, affections, appetites, and passions. It is something greater than a mere thinking machine that is required to guide and regenerate this blurred image of the Eternal."

These statements are eloquent from their inherent truth and importance. Judges may administer the laws, but their knowledge of physiology is often as defective as that of the juries who have to decide on the guilt or innocence of those who may be under trial.

And we have often observed that their psychological information is also often defective, which has been shown in some of the articles in this journal. For instance, in that able one by its learned Editor on the case of Luigi Buranelli, in which it is clearly shown that that unfortunate foreigner was insane when he committed the act, and that he was more fitted for an asylum than to be harassed with a trial, and to suffer the last penalty of the law.

It may be difficult always to draw the line of demarcation between crime and insanity, in both of which there is defective moral liberty, yet Dr. Forbes Winslow has done this in the above cited case. This important distinction has also been attempted by our legislators, as in Lord Ellenborough's Act; in which it is laid down in extenuation of homicide, that if one man kills another under some sudden burst of passion, without any *malice prepense*, and without having had time to deliberate on his act, that it is to be regarded as manslaughter.

If, then, our views of moral liberty are admitted, we have in the case of criminals some data for their improvement. For we consider the conditions for perfect moral liberty, the acting under the *dicta* of the intellectual and moral attributes, and the relative degree must depend on the positive or comparative excellence of the natural powers, and of their more or less judicious culture.

For to ensure physical health we must obey the hygienic laws; and so also, unless we obey the physiological and psychological conditions on which the mind's sanity depends, evil must necessarily result, and as a consequence, crime is ultimately generated.

For example, there cannot be a large number of men and women of neglected education and debased habits without their becoming a festering mass, polluting the very atmosphere, until the moral health of domestic servants, shopmen, confidential clerks, &c., is more or less affected with the consequences. The remedy is education. Not merely teaching reading and writing, which are not even the rudiments of knowledge, but the mere instruments for collecting, arranging, and preserving materials for thought. We must train the moral attributes, and regulate the feelings, so that every person in the community may know how to control their actions, and this not from any fear of punishment for infringing the laws, but from a conviction that there is always a great advantage to individuals in obeying the higher motives of the mind.

In the absence of the means of positive demonstration of this important problem, we may by an inductive process draw some satisfactory inference by comparing one thing with another. It is admitted, for instance, that before there can be symmetry of body, its different parts must be trained and exercised according to fixed laws; and so it is with the mental faculties. For the greater portion of the evils which now blight the fair prospects of society spring from the abuse or the defective condition of moral liberty.

What then can be said in apology for our dooming a large portion of the community to a state of actual and deplorable ignorance, which leaves them subject to mere sensual excitements, the natural *stimuli* of crimes, and then marvel that the consequent result is that they are guilty of criminal actions. With what grace can we afterwards inflict on such beings certain penalties, when we ourselves have heartlessly deprived them of the only means of self-control? If we would bestir ourselves, and spend the money in preventing crimes by moral means that we are now forced to pay to punish the hordes of offenders who prey upon us, we may remedy the evil, but who must, by an inevitable law, without some active means of reformation, ultimately induce more certain and general ruin, by involving the very elements of society in a state of chaos, where all will be once more "without form or order," and an ominous darkness rest over the length and breadth of the community. L.

